# Comparative efficacy of modified FOLFIRINOX, gemcitabine plus capecitabine and gemcitabine plus nab-paclitaxel as adjuvant treatment for resected pancreatic cancer: a Bayeasian network meta-analysis

**DOI:** 10.3332/ecancer.2021.1305

**Published:** 2021-10-12

**Authors:** Victor Hugo Fonseca de Jesus, Rachel P Riechelmann

**Affiliations:** Medical Oncology Department, A.C. Camargo Cancer Center, Rua Prof. Antônio Prudente 211, São Paulo SP 01509-010, Brazil

**Keywords:** FOLFIRINOX, gemcitabine, capecitabine, nab-paclitaxel, adjuvant, pancreatic, cancer

## Funding source

This study had no funding source.

## Authors’ contributions

Victor Hugo Fonseca de Jesus: conceptualisation, methodology, data curation, data analyses, writing and visualisation; Rachel Pimenta Riechelmann: methodology, writing and visualisation.

